# Supplementary material for: MicroRNA Sequencing Revealed Citrus Adaptation to Long-Term Boron Toxicity through Modulation of Root Development by miR319 and miR171
Source: Int J Mol Sci. 2019 Mar 21;20(6):1422. doi: 10.3390/ijms20061422 (PMC6470687; doi:10.3390/ijms20061422)
Supplement: Supplementary file 1 [file ijms-20-01422-s001.zip › Supplementary Figures.docx]

**Supplementary Figures:**


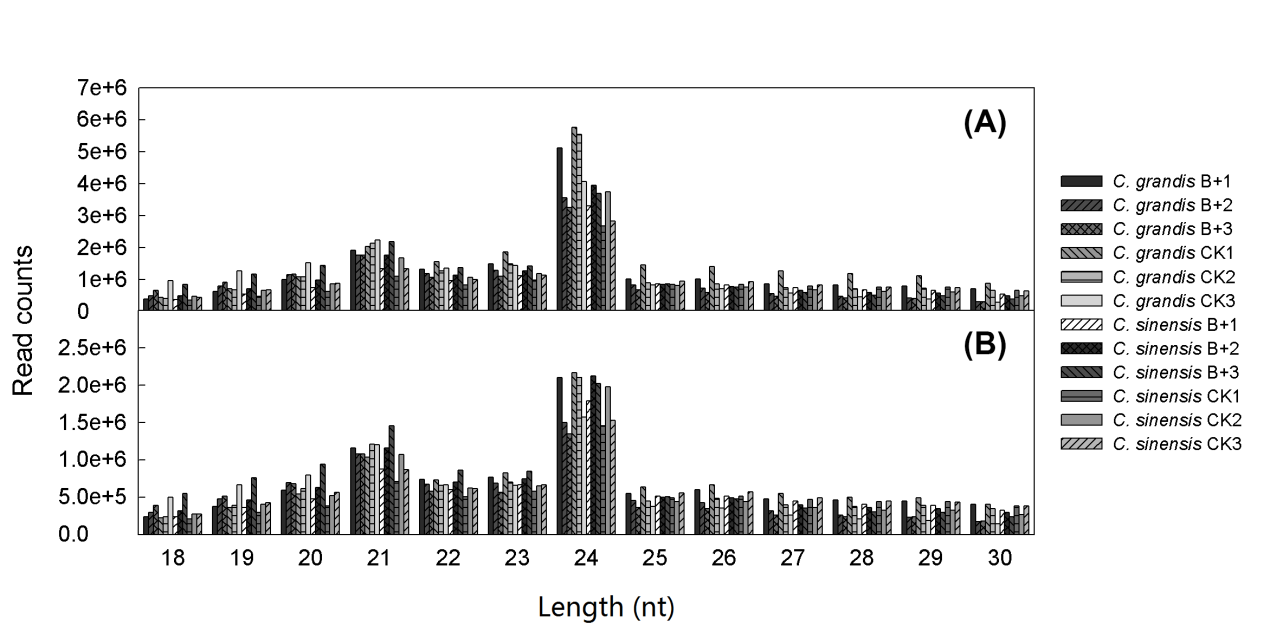


Figure S1. Size distributions of sRNAs in the 12 libraries of *Citrus* root tips treated with different B levels. (A) Length distribution of clean reads, (B) number of clean reads mapped to the *C. clementina* genome.


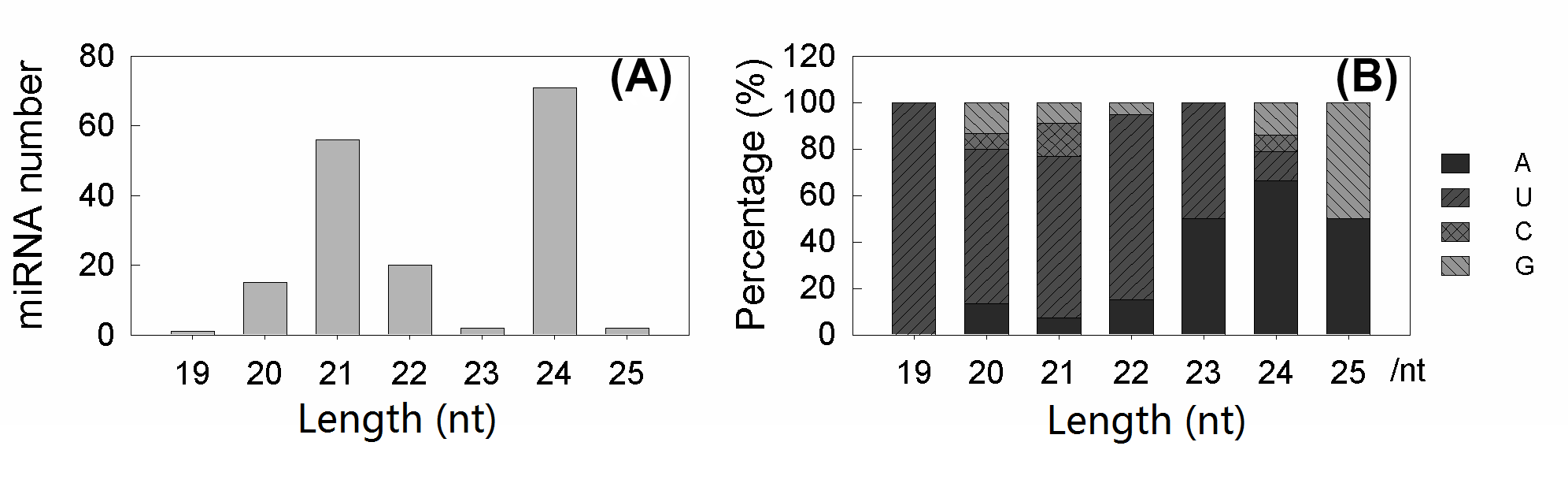


Figure S2. Size distributions (A) and first nucleotide bias of candidate miRNAs (B) in *Citrus* root tips.


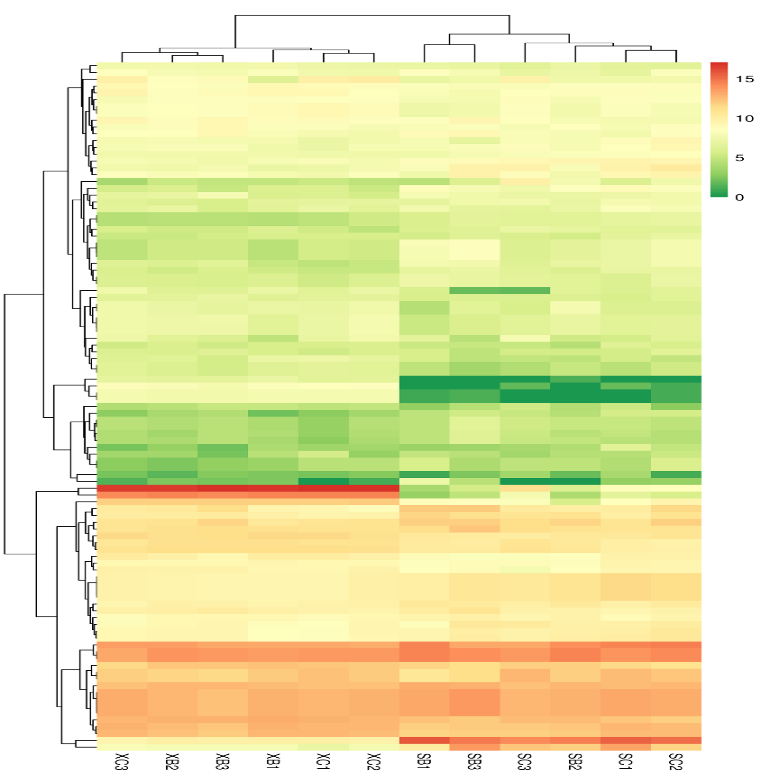


Figure S3. DEG cluster of DE miRNAs in *Citrus* root tips treated with different B level. XC, Control *C. sinensis*; XB, B-toxic *C. sinensis*; SC, Control *C. grandis*; SB, B-toxic *C. grandis*.


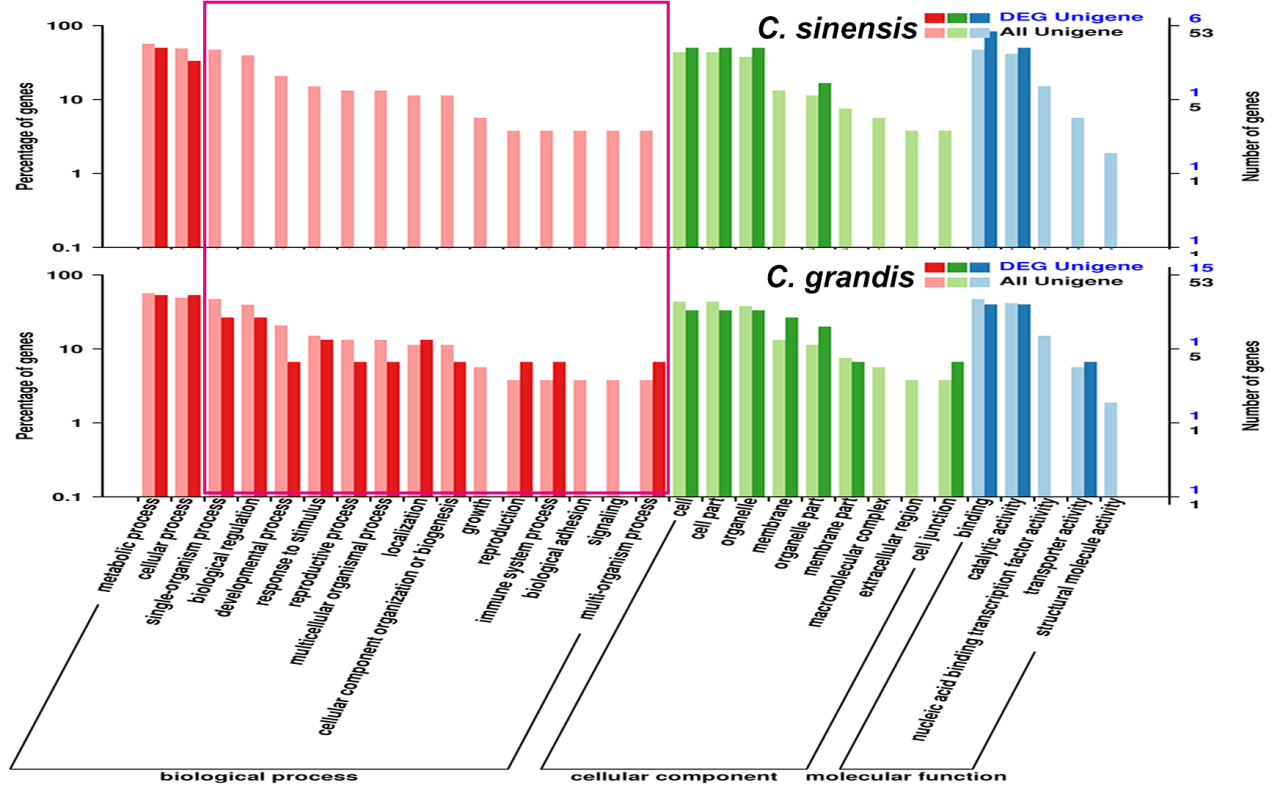


Figure 4. GO analyses of predicted target genes from DE miRNAs under B-toxic stress in *Citrus*. The pink rectangle indicated significant changes of sample frequencyies (the dark bars) of GO terms enriched between the two species. The light bars displayed background frequencies of GO terms enriched in putative B-toxic ressponsive genes targeted by DE miRNAs.
