# Supplementary material for: MicroRNA Sequencing Revealed Citrus Adaptation to Long-Term Boron Toxicity through Modulation of Root Development by miR319 and miR171
Source: Int J Mol Sci. 2019 Mar 21;20(6):1422. doi: 10.3390/ijms20061422 (PMC6470687; doi:10.3390/ijms20061422)
Supplement: Supplementary file 1 [file ijms-20-01422-s001.zip › Supplementary Methods.docx]

**Supplementary methods**

***QRT-PCR of differentially expressed miRNAs and candidate targets***

For validation of differentially expressed (DE) miRNAs obtained from Illumina sequencing, 22 miRNAs were randomly selected to perform stem-loop qRT-PCR using *U6* snRNA as an internal control (Xie et al., 2017). Stem-loop RT primers and miRNA-specific primers (forward) were designed as previously reported by Huang et al. (Huang et al., 2016) (Supplementary Table 1). Root total RNA of each replicate (1 μg) was used to create a reverse transcription pool with the TaqMan® MicroRNA Assay Kit (Takara, Japan) according to the manufacturer’s instructions. qRT-PCR was then performed as described previously (Huang et al., 2016), using the miRNA-specific forward primer and a Universal Reverse Primer (Supplementary Table 1). The cycling conditions were set as follows: 10 min at 95 ℃, followed by 40 cycles of 95 ℃ for 15 s and 60 ℃ for 60 s.

To monitor the relative abundance of candidate targets, gene-specific forward and reverse primers were designed using Primer Software (PREMIER Biosoft International, U.S.A., version 5.0). The forward and reverse primers were approximately 200 bp up- and down-stream from potential cleavage sites, respectively, producing an expected fragment of approximately 100-300 bp in size (Supplementary Table 2). qRT-PCR reactions were performed on an Mastercycler Ep Realplex System (Eppendorf, Germany), 95 °C for 10 min, 40 cycles at 95 °C for 15 s, 60 °C for 15 s, and 72 °C for 15 s, using *actin* (AEK97331.1) as an internal control.

As total RNA was used for reverse transcription, melting curve analysis followed by agarose gel electrophoresis was conducted to determine the purity and fragment size of the amplicons. The relative expression levels of miRNAs and candidate targets were determined by the 2^−∆∆Ct^ method. Results were then analysed by IBM SPSS statistics software (Version 22).

**References**

Huang, J.H., Qi, Y.P., Wen, S.X., Guo, P., Chen, X.M., Chen, L.S., 2016. Illumina microRNA profiles reveal the involvement of miR397a in Citrus adaptation to long-term boron toxicity via modulating secondary cell-wall biosynthesis. Sci Rep 6, 22900.

Xie, R., Zhang, J., Ma, Y., Pan, X., Dong, C., Pang, S., He, S., Deng, L., Yi, S., Zheng, Y., Lv, Q., 2017. Combined analysis of mRNA and miRNA identifies dehydration and salinity responsive key molecular players in citrus roots. Sci Rep 7, 42094.
